# Parent-reported and clinician-observed autism spectrum disorder (ASD) symptoms in children with attention deficit/hyperactivity disorder (ADHD): implications for practice under DSM-5

**DOI:** 10.1186/s13229-016-0072-1

**Published:** 2016-01-19

**Authors:** Rebecca Grzadzinski, Catherine Dick, Catherine Lord, Somer Bishop

**Affiliations:** Center for Autism and the Developing Brain, Weill Cornell Medical College, NewYork-Presbyterian Hospital, 21 Bloomingdale Road, White Plains, NY 10605 USA; Teachers College, Columbia University, 525 West 120th Street, New York, NY 10027 USA; School of Medicine, University of California, San Francisco, 401 Parnassus Avenue, San Francisco, CA 94143 USA

**Keywords:** Autism, Autism Spectrum Disorder (ASD), Attention Deficit/Hyperactivity Disorder (ADHD), Autism Diagnostic Observation Schedule (ADOS), Autism Diagnostic Interview-Revised (ADI-R), Diagnostic and Statistical Manual of Mental Disorders, Fifth Edition (DSM-5)

## Abstract

**Background:**

Children with attention deficit/hyperactivity disorder (ADHD) often present with social difficulties, though the extent to which these clearly overlap with symptoms of autism spectrum disorder (ASD) is not well understood.

**Methods:**

We explored parent-reported and directly-observed ASD symptoms on the Autism Diagnostic Interview-Revised (ADI-R) and the Autism Diagnostic Observation Schedule (ADOS) in children referred to ASD-specialty clinics who received diagnoses of either ADHD (*n* = 48) or ASD (*n* = 164).

**Results:**

Of the ADHD sample, 21 % met ASD cut-offs on the ADOS and 30 % met ASD cut-offs on all domains of the ADI-R. Four social communication ADOS items (Quality of Social Overtures, Unusual Eye Contact, Facial Expressions Directed to Examiner, and Amount of Reciprocal Social Communication) adequately differentiated the groups while none of the items on the ADI-R met the criteria for adequate discrimination.

**Conclusions:**

Results of this work highlight the challenges that clinicians and researchers face when distinguishing ASD from other disorders in verbally fluent, school-age children.

## Background

Autism Spectrum Disorder (ASD) and Attention Deficit/Hyperactivity Disorder (ADHD) are two of the most commonly diagnosed childhood neurodevelopmental disorders [1]. As described in the *Diagnostic and Statistical Manual of Mental Disorders*, *Fifth Edition* (DSM-5) [1], core symptoms of an ASD diagnosis do not overlap with core symptoms of ADHD, though research suggests that 30–75 % of children with a diagnosis of ASD have symptoms of ADHD (see [2–7]) and 20–60 % of children with ADHD have ASD-like social difficulties (see [8–12]). Over the last several years, there has been increasing interest in ASD-ADHD symptom overlap [13]. This led to the removal in DSM-5 of the *Diagnostic and Statistical Manual of Mental Disorders*, *Fourth Edition*, *Text Revision* (DSM-IV-TR) restriction that prevented a dual diagnosis of ASD and ADHD. This provides clinicians with an opportunity to acknowledge both the converging and discrete symptom presentations of ASD and ADHD. It also underscores the importance of understanding the boundaries and overlaps between these disorders [14].

One approach to achieve a better understanding of the convergence between the two disorders is through exploration of ASD symptoms in individuals with a diagnosis of ADHD. Research to date has found that social dysfunction occurs regularly in children with ADHD [5, 15–17] and these social deficits may be similar to those seen in ASD [9, 10, 18]. One study described ASD symptoms in children with ADHD as loading on two factors, social communication and restricted, repetitive behaviors [19], the same way that these constructs have been shown to organize in ASD samples [20, 21]. Other work has shown that ASD symptoms are elevated in children with more behavioral difficulties, including ADHD symptoms [22–25]. Similarly, symptoms of ASD have been found to be associated with oppositional behaviors and depression in children with ADHD [8, 9, 11]. Thus, it remains unclear whether ASD-like social difficulties in ADHD are fully explained by associated behavioral or mood symptoms or whether behavior/mood difficulties *and* ASD-like symptoms occur separately in ADHD and lead to social dysfunction.

One limitation of research exploring ASD symptoms in individuals with ADHD is that the majority of studies have relied on parent questionnaires of ASD symptoms and/or measures of broad psychopathology that are not specific to ASD [5, 8–11, 16–18, 26, 27]. None of these studies used direct child observation measures, such as the Autism Diagnostic Observation Schedule (ADOS) [28], or an ASD-specific parent interview, such as the Autism Diagnostic Interview, Revised (ADI-R) [29], to characterize ASD symptoms in children with ADHD. The ADOS and the ADI-R are two of the most widely used and well-validated measures designed specifically to assess ASD symptoms. The ADOS and ADI-R provide detailed information about specific social and repetitive behaviors while also providing empirically defined diagnostic domain scores. In addition, items on the ADOS and ADI-R have been mapped to DSM-5 diagnostic criteria for ASD [30], providing an opportunity to inform the practical application of DSM-5 criteria in children with ADHD and ASD symptoms. Although current research motivations emphasize exploration of symptoms on a continuum (Research Domain Criteria (RDoC)) [31], current clinical needs require practitioners to provide dichotomous determinations of whether someone does or does not have a disorder. Therefore, exploring the prevalence of specific ASD symptoms in children with ADHD has important implications for clinicians seeking diagnostic clarification. In addition, when looking at domains of symptoms on ASD measures in a continuous fashion, we would expect that the children with ASD diagnoses would have more severe symptoms than children with ADHD; however, clinicians may be particularly guided by a better understanding of which specific symptoms are most discriminative between groups. Furthermore, obtaining detailed information about specific social difficulties in children with ADHD has implications for treatment planning, as well as for interpreting emerging evidence of both genetic [19, 32] and neurobiological [33–35] overlap between these disorders.

In this study, we examined ADOS and ADI-R scores in a sample of children referred to ASD-specialty clinics for diagnostic evaluations who ultimately received clinical diagnoses of ADHD (not ASD). This is a unique sample given that most comparative work between ASD and ADHD has used research rather than clinical samples; this particular group of children may be especially relevant to clinicians. We aimed to explore (1) the specificity of standard ASD diagnostic instruments in children referred to an ASD clinic who received a diagnosis of ADHD and (2) the specific profiles of ASD symptoms (on a domain and individual item level) in this sample of children with ADHD compared to age, sex, and IQ-matched children with primary diagnoses of ASD.

## Methods

### Participants

Data from the current study were extracted from an existing database of children whose parents had provided written informed consent for their child’s clinical information to be included in a research database which was approved by Institutional Review Boards (IRB) at three different ASD-specialty clinics (sites): the University of Chicago Developmental Disorders Clinic (UCDDC; 45 % of the sample), the University of Michigan Autism and Communication Disorders Center (UMACC; 47 % of the sample), or the New York Presbyterian Center for Autism and the Developing Brain (CADB) at Weill Cornell Medicine (8 % of the sample). Children were only eligible for the current study if they had been referred for a clinical ASD diagnostic assessment (i.e., not seen as part of a specific research study) and had received an overall best estimate clinical diagnosis (BEC) of ASD or ADHD following the comprehensive assessment (see below).

Research has shown that distinguishing between ASD and ADHD in children who are verbally and intellectually able is a particular challenge [18, 36, 37]. Therefore, in this initial exploration, we limited our sample to children who were between the ages of 4 and 18 years old, with IQs in the borderline range or above (full scale IQs ≥70), and received module 3 or 4 of the ADOS [38], indicating verbal fluency. Forty-eight participants (38 males) who received clinical assessments and met the above criteria received a BEC of ADHD (no diagnosis of ASD). Children with a BEC of ASD were chosen as a comparison group, based on the same criteria outlined above. Due to the DSM-IV-TR prohibition against diagnosis of comorbid ADHD in individuals with ASD, ADHD was not systematically ruled in/out in children with ASD prior to 2013 (when DSM-5 criteria were implemented); however, children who were given a comorbid diagnosis of ASD *and* ADHD (post-DSM-5 implementation; *n* = 66) were not eligible for inclusion in this study. The final sample of children with a BEC of ASD included 164 children (138 males). Information about current psychotropic medication treatment was only available for 66 % (*n* = 139) of the sample, as this information was not systematically gathered for children seen at UCDDC. There was no statistically significant difference between the ADHD and ASD groups with regard to the proportion of children receiving psychotropic medication (*χ*^2^(1, 139) = 3.36, *p* = 0.07). The children with ADHD did not differ from children with ASD in verbal IQ (VIQ), non-verbal IQ (NVIQ), age, gender, or ethnicity. However, given the large age (4 to 18 years old) and NVIQ (72–153) ranges included in the study, these variables were included as covariates in secondary analyses of dimensional data despite no significant overall group differences in means. In addition, because significantly more children with ADHD were seen in Michigan than New York and Chicago, site was included as a covariate in these secondary analyses (see Table 1).

**Table 1:** Descriptive data for ADHD and ASD samples

	ADHD	ASD			
	(*n* = 48)	(*n* = 164)			
	Mean (SD)	Mean (SD)	*t*	*df*	*p* value
Age (years)	9 (4)	9 (3)	0.01	210	0.99
VIQ^a^	106 (16)	103 (18)	0.75	208	0.45
NVIQ^a^	105 (14)	104 (17)	0.21	207	0.83
	*n* (%)	*n* (%)	*χ* ^2^	*df*	*p* value
Males	38 (79)	138 (84)	0.65	1	0.42
ADOS Mod 3	41 (85)	145 (88)	0.31	1	0.58
Site					
UMACC	29 (60)	70 (43)	26.90	2	<0.001
UCDDC	9 (19)	88 (54)			
CADB	10 (21)	6 (4)			

*ADHD* attention deficit/hyperactivity disorder, *ASD* autism spectrum disorder, *CADB* Center for Autism and the Developing Brain in New York, *df* degrees of freedom, *UCDDC* University of Chicago Developmental Disorders Clinic, *UMACC* University of Michigan Autism and Communications Disorders Center

^a^Two ADHD participants were missing VIQ information and three additional ADHD participants were missing NVIQ information

### Measures

#### ASD measures

The ADOS [28] and the ADI-R [29] were administered in order to obtain information about ASD symptoms. The ADI-R is a standardized parent interview that obtains information about past and current symptoms of ASD. The ADOS obtains information through direct observation. All clinicians involved in administering these instruments had established research reliability. Items on the ADOS and ADI-R are scored on a 0–3 scale, with higher scores indicating greater impairment. Per ADOS and ADI-R algorithm scoring conventions, all scores of 3 were converted to 2.

The ADI-R yields four domain scores based on past behavior (between the ages of 4 and 5, or ever), which are calculated by summing items within the areas of Qualitative Abnormalities in Reciprocal Social Interaction (Social), Qualitative Abnormalities in Communication (Communication), Restricted, Repetitive, and Stereotyped Patterns of Behavior (RRB-ADI), and Abnormality of Development Evident at or Before 36 Months (Age of Onset). Totals from domains have algorithm cut-offs that yield classifications of autism or non-autism.

In addition, in order to understand current behavior described on the ADI-R, domains of social communication (SC), repetitive sensory motor (RSM), and insistence on sameness (IS) were calculated using domains defined by Lord, Bishop, and Anderson [38]. Because all participants were verbally fluent in our sample, we included verbal items from the ADI-R that Lord, Bishop, and Anderson had excluded [38]; items 34–38 (Social Verbalization/Chat, Reciprocal Conversation, Inappropriate Questions/Statements, Pronominal Reversal, and Neologisms/Idiosyncratic Language) were placed in the SC domain, item 33 (Stereotyped Utterances/Delayed Echolalia) was placed in the RSM domain, and items 39 (Verbal Rituals) and 68 (Circumscribed Interests) were placed in the IS domain.

The ADOS diagnostic algorithm yields classifications of ASD versus non-ASD [39, 40] as well as Calibrated Severity Scores (CSS) for the algorithm total (CSS Overall) and domain severity scores in the areas of social affect (CSS SA) and restricted and repetitive behavior (CSS RRB) [40, 41]. In addition, ADOS totals were calculated in the domains of basic social communication (basic SOC), interaction quality, and RRBs based on recently defined ADOS factors (see [42]).

To aid in diagnostic symptom understanding, researchers have mapped individual items from the ADOS and ADI-R to ASD symptom domains [30]. For the purposes of this study, to be most consistent with diagnostic domains, the ADOS item Amount of Reciprocal Social Communication, which was originally mapped by Huerta et al. [30] to symptom area “Developing and maintaining relationships” (A3) [30], was moved to symptom area “Socio-emotional reciprocity” (A1).

### Cognitive testing

For the majority of participants, a standard hierarchy was followed to select appropriate cognitive tests: the Differential Ability Scales (DAS) [43] was chosen first, followed by an age-appropriate Wechsler test (Wechsler Adult Intelligence Scale (WAIS), Wechsler Abbreviated Scale of Intelligence (WASI), Wechsler Intelligence Scale for Children (WISC), Wechsler Preschool and Primary Scale of Intelligence (WPPSI)) [44–47]. These tests provided verbal IQ (VIQ) and non-verbal IQ (NVIQ) for 93 % and 85 % of the ADHD and ASD groups, respectively. These measures use chronological age standardization with a mean of 100 and a standard deviation of 15 and have adequate convergent validity [45]. A number of other tests were used to estimate IQ for the remaining participants (7 % of the ADHD sample and 15 % of the ASD sample). Specifically, approximations of VIQ were gathered from the *Clinical Evaluation of Language Fundamentals* (CELF) [48], Peabody Picture Vocabulary Test (PPVT) [49], Vineland Adaptive Behavior Scales (VABS) [50], and NVIQ from the Raven’s Progressive Matrices [51]. There was no difference between the diagnostic groups with regards to which cognitive test was used.

### Clinical diagnosis

An overall best estimate clinical diagnosis (BEC) [52] was determined based on all available information, including questionnaires, information from teachers, results of the ADOS and ADI-R, and the diagnostic impression of a clinical team, which included a psychologist and/or psychiatrist.

### Analysis

First, we examined the proportion of participants who met standard ADI-R and ADOS algorithm cut-offs in the ADHD group. We expanded this to also explore less strict ADI-R cut-offs for ASD as defined by Lainhart et al. (Collaborative Program of Excellence in Autism (CPEA) ADI-R Criteria) [53] since original ADI-R cut-offs were intended to capture autistic disorder, with less sensitivity to broader ASD presentations now captured under DSM-5 criteria. Second, using analysis of variance (ANOVA), the groups were compared on ADOS CSS (Overall, SA, and RRB), ADOS factors (basic SOC, interaction quality, and RRBs), ADI-R algorithm domains (social, communication, RRB-ADI), and ADI-R current symptom domains (SC, RSM, and IS). Secondary analyses of covariance (ANCOVA) co-varying for age, NVIQ, and site were conducted. The ADI-R age of onset algorithm domain is intended for categorical use; therefore, a chi-square analysis was conducted comparing groups on the proportion of children who met the domain algorithm cut-off (≥1). Third, since current diagnostic practice (DSM-5) focuses primarily on the presence/absence of specific symptoms and since ADI-R and ADOS items represent ordinal rather than interval data, we compared the two groups with regard to the proportion of symptoms endorsed (1, 2, or 3) versus not endorsed (0). Specifically, *χ*^2^ analyses were conducted to compare proportions of endorsed versus non-endorsed items that have been mapped to each symptom domain [30]. Fourth, using methodology previously employed for the purpose of identifying items for diagnostic algorithms and other discrimination purposes [39, 54, 55], we identified specific social communication items that discriminated best between the groups. This methodology aims to identify items that show both a shifted distribution by diagnostic group and also items that are most likely to differentiate between ASD and non-ASD. An item was determined to discriminate “adequately” between groups if it was endorsed (code of 1–3) in >66 % of the ASD group and <33 % of the ADHD group. This criterion was not applied to RRB items because endorsement was not expected in a large proportion (>66 %) of individuals with ASD [39], especially given that participants in this study were primarily school-aged and adolescent children with average or above IQ. To account for multiple comparisons, a significance threshold was set at *α* = 0.01 across analyses.

## Results

### Specificity of the ADI-R and ADOS

As Table 2 shows, 21 % of the ADHD sample and 85 % of the ASD sample met ADOS ASD algorithm cut-offs (cut-off for ASD ≥7 for module 3 and ≥8 for module 4) [39, 40]. On the ADI-R, 30 % of the ADHD sample and 67 % of the ASD sample met algorithm criteria across all four domains resulting in an ADI-R classification of autism. When using CPEA criteria, which are less strict than standard ADI-R autism cut-offs [53], 67 % of the children with ADHD and 84 % of the ASD sample received an ADI-R classification of ASD. When the measures were employed in combination, most children (60–75 %) with ASD met criteria for ASD on both the ADOS and the ADI-R, while few children with ADHD met criteria for ASD on both the ADOS and the ADI-R (11 % when using traditional ADI-R cut-offs and 15 % when using CPEA ADI-R cut-offs).

**Table 2:** Percent meeting cut-offs on the ADOS and ADI-R

	ADHD	ASD
	(*n* = 48)^a^	(*n* = 164)^a^
ADOS cut-off	21	85
ADI-R (social, communication, RRB, and age of onset) cut-offs	30	67
Social cut-off	57	82
Communication cut-off	61	87
RRB cut-off	67	88
Age of onset cut-off	80	88
ADI-R CPEA ASD criteria^a^	67	84
Combining measures		
ADOS and ADI-R (social, communication, RRB, and age of onset)	11	60
ADOS and ADI-R CPEA ASD criteria^a^	15	75

*ADHD* attention deficit/hyperactivity disorder, *ASD* autism spectrum disorder, *df* degrees of freedom, *RRB* restricted repetitive behaviors on ADI-R

^a^CPEA criteria was determined based on Lainhart et al. [53]; Two children with ADHD and eight children with ASD were missing ADI-R data

### Domain comparisons

#### ADOS

**Fig. 1:**
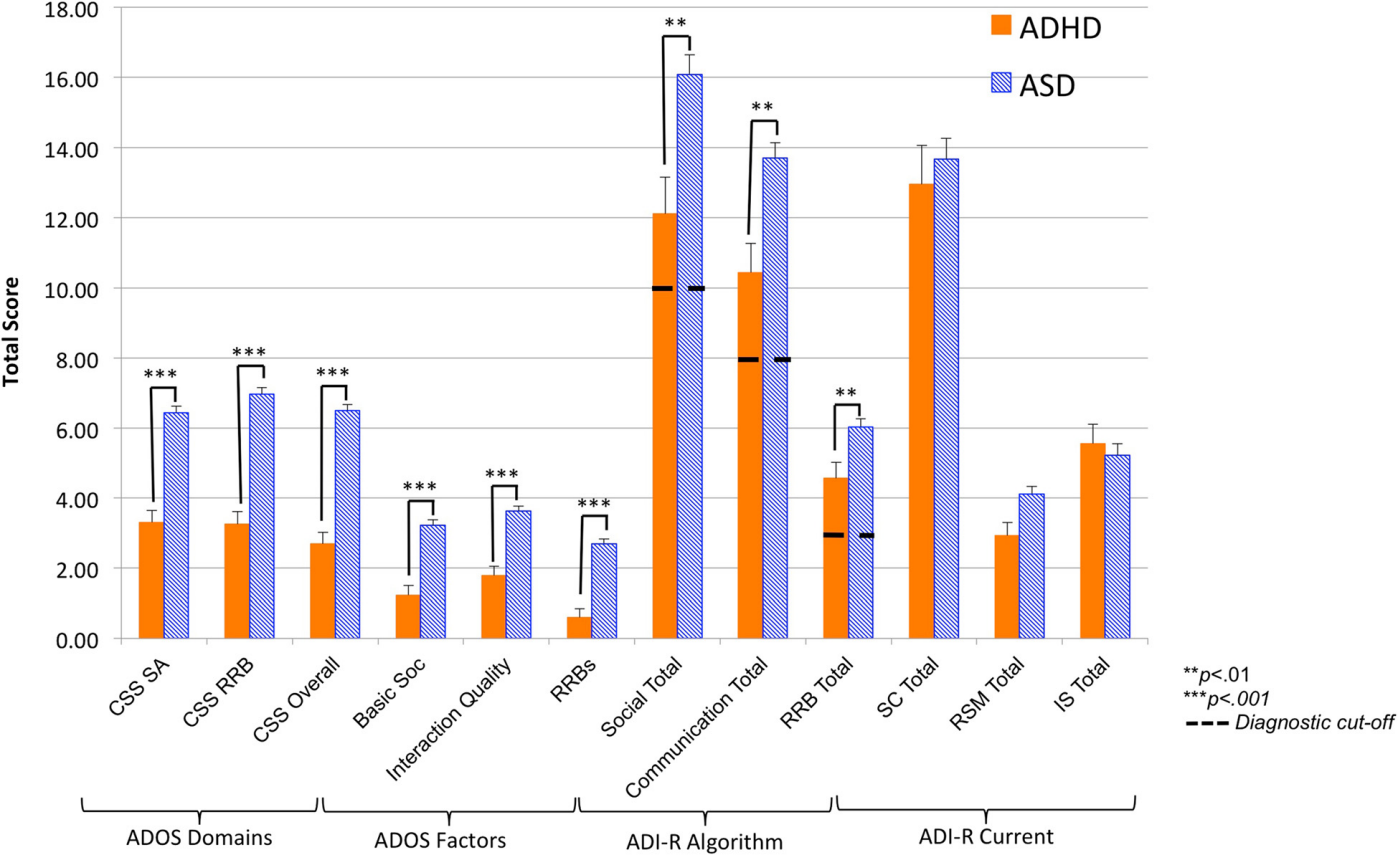
ADOS and ADI-R domain scores. *ADHD* attention deficit/hyperactivity disorder, *ASD* autism spectrum disorder, *CSS* calibrated severity score, *IS* insistence on sameness, *RRBs* restricted repetitive behaviors, *RSM* repetitive sensory motor, *SA* social affect, *SC* social communication. Results presented do not include covariates; see text for results of analyses of covariance

Not surprisingly, the ASD group scored significantly higher than the ADHD group on all CSS domains (CSS SA, CSS RRB, and CSS Overall): *F*(1,210) = 66.80, *p* < 0.001, *η*^2^ = 0.24, *F*(1,210) = 80.60, *p* < 0.001, *η*^2^ = 0.28, and *F*(1,210) = 99.51, *p* < 0.001, *η*^2^ = 0.32, respectively. The ASD group also scored significantly higher on basic SOC, interaction quality, and RRBs factors [42]: *F*(1,201) = 41.16, *p* < 0.001, *η*^2^ = 0.17, *F*(1,210) = 38.19, *p* < 0.001, *η*^2^ = 0.15, *F*(1,210) = 53.26, *p* < 0.001, *η*^2^ = 0.20, respectively (see Fig. 1 and Appendix: Table 6). These results remained consistent when controlling for site, age, and NVIQ.

#### ADI-R

Children with ASD had significantly higher scores on the social, communication, and RRB domains of the ADI-R: *F*(1,200) = 11.43, *p* < 0.01, *η*^2^ = 0.05, *F*(1,200) = 12.24, *p* < 0.01, *η*^2^ = 0.06, and *F*(1,200) = 8.22, *p* < 0. 01, *η*^2^ = 0.04. Analyses indicated that there was no statistically significant difference between groups in terms of the proportion of those who met the age of onset domain cut-off. There were no statistically significant differences between the groups across current symptom domains on the ADI-R (SC, RSM, and IS as defined by [38]). These results remained consistent when controlling for age, NVIQ, and site with one exception: when controlling for age, a group difference in the RSM total (current) of the ADI-R emerged: *F*(2,192) = 7.01, *p* < 0. 01, *η*^2^ = 0.04. See Fig. 1 and Appendix: Table 6.

### Individual items/symptoms

#### Social-emotional reciprocity (symptom area A1; seven ADOS items, seven ADI-R items)

Results of *χ*^2^ tests of differences in proportion indicate that all ADOS items in the symptom area of social-emotional reciprocity (A1), except Shared Enjoyment in Interaction and Asks for Information, were endorsed significantly more frequently in the ASD group than the ADHD group (see Table 3). Two of these items also met study criteria as adequate discriminators: Quality of Social Overtures and Amount of Reciprocal Social Communication (see Table 3). Few ADI-R items were endorsed significantly more frequently in the ASD group than the ADHD group and none met study criteria as Individual items/symptoms (see Table 4).

**Table 3:** Percent endorsed ADOS item abnormality (non-zero scores) by diagnosis

	ADHD	ASD
	(*n* = 48)	(*n* = 164)
A1. Social-emotional reciprocity		
Quality of social overtures (item B7/B9)***	*27*	*70*
Shared enjoyment in interaction (item B4)	17	38
Quality of social response (item B9/B11)**	56	80
Conversation (item A8)***	50	80
Offers information (item A5)**	13	41
Asks for information (item A6)	77	86
Amount of reciprocal social communication (item B10/B12)***	*25*	*68*
A2. Non-verbal communicative behaviors		
Unusual eye contact (item B1)***	*31*	*71*
Facial expressions directed to examiner (item B2)***	*13*	*67*
Descriptive, conventional, instrumental, or informational gestures (item A9)	29	46
B1. Stereotyped/repetitive speech, motor movements, or use of objects		
Immediate echolalia (item A3)	2	15
Stereotyped/idiosyncratic use of words/phrases (item A4)***	31	71
Hand and finger and other complex mannerisms (item D2)**	2	30
B2. Excessive adherence to routines, ritualized patterns, or resistance to change		
Compulsions or rituals (item D5)	21	27
B3. Highly restricted, fixated interests		
Excessive Interest in or References to Unusual or Highly Specific Topics or Objects or Repetitive Behaviors (item D4)***	15	55
B4. Hyper-/hypo-reactivity to sensory input or unusual interest in sensory environment		
Unusual sensory interest in play material/person (item D1)**	10	34

Based on Huerta, Bishop, Duncan, Hus, and Lord, 2012 item classification for DSM-5; items in italics are those that met criteria for adequacy at distinguishing between groups (endorsed in >66 % of ASD and <33 % of ADHD)

**p < 0.01, ***p < 0.001

#### Non-verbal communicative behaviors (symptom area A2; three ADOS items, six ADI-R items)

In the symptom area of non-verbal communication (A2), two of the ADOS items were endorsed significantly more frequently in the ASD group (*χ*^2^ tests of differences in proportion) and also met study criteria as adequate discriminators: Unusual Eye Contact and Facial Expressions Directed to Examiner (see Tables 3 and 5). No statistically significant differences between groups in the proportion of endorsed ADI-R items were found, and none of the ADI-R items emerged as adequate discriminators (see Table 4).

**Table 4:** Percent endorsed ADI-R DSM-5 diagnostic item abnormality (non-zero scores) by diagnosis

	ADHD	ASD
	Past (current)	Past (current)
A1. Socio-emotional reciprocity		
Social chat (item 34)	71 (61)	79 (67)
Conversation (item 35)**	69 (70)	90 (88)
Use of other’s body to communicate (item 31)^^	15 (13)	24 (7)
Showing and directing attention (item 52)	35 (24)	57 (36)
Seeking to share enjoyment (item 54)	33 (25)	55 (31)
Offering comfort (item 55)	49 (47)	69 (55)
Social smiling (item 51)	58 (51)	73 (66)
A2. Non-verbal communicative behaviors		
Pointing (item 42)	52 (50)	67 (56)
Nodding (item 43)	41 (44)^a^	45 (32)
Shaking (item 44)	30 (40)	40 (32)
Conventional/instrumental gestures (item 45)	51 (44)	75 (63)
Range of facial expressions (item 57)	60 (53)	66 (60)
Quality of social overture (item 56)	52 (49)	65 (49)
A3. Developing and maintaining relationships		
Inappropriate questions (item 36)	67 (69)^a^	67 (65)
Inappropriate facial expressions (item 58)	54 (56)^a^	57 (54)
Offering to share (item 53)	72 (64)	74 (63)
Appropriateness of social responses (item 59)	61 (64)^a^	79 (73)
Social disinhibition (item 66)	84 (82)	83 (80)
Friendships (item 65)^b^	64 (73)	82 (79)
B1. Stereotyped/repetitive speech, motor movements, or use of objects		
Stereotyped language (item 33)***	52 (47)	72 (68)
Pronoun reversal (item 37)	28 (11)	38 (14)
Neologisms/idiosyncratic language (item 38)	26 (24)	34 (21)
Repetitive use of objects (item 69)	61 (47)	65 (46)
Hand/finger mannerisms (item 77)	20 (18)	40 (34)
Other complex mannerisms (item 78)	28 (22)	50 (43)
B2. Excessive adherence to routines, ritualized patterns, or resistance to change.		
Verbal rituals (item 39)	33 (33)	38 (33)
Compulsions/rituals (item 70)	48 (42)	54 (49)
Difficulties with minor changes in routines/environment (item 74)	66 (66)	69 (61)
Resistance to trivial changes in the environment (item 75)	24 (22)	19 (16)
B3. Highly restricted, fixated interests.		
Unusual preoccupations (item 67)	26 (22)	38 (29)
Circumscribed interests (item 68)	67 (66)	76 (71)
Unusual attachment to objects (item 76)	24 (16)	29 (15)
B4. Hyper-/hypo-reactivity to sensory input or unusual interest in sensory environment		
Undue general sensitivity to noise (item 72)	50 (48)	69 (63)
Abnormal, idiosyncratic, negative response to specific sensory stimuli (item 73)	53 (55)	63 (61)
Unusual sensory interests (item 71)	61 (64)^b^	63 (57)

Based on Huerta, Bishop, Duncan, Hus, and Lord, 2012 item classification for DSM-5

^a^Two parents of ADHD children and eight parents of ASD children were not administered the ADI-R and one additional child with ADHD was not administered current items; therefore, these individuals were not included in analyses and percentages; higher percentage of current versus past explained by missing current data

^b^Friendships (item 65) was not originally mapped to DSM-5 criteria by Huerta, Bishop, Duncan, Hus, and Lord, 2012 but was added here due to its applicability to the symptoms area; in addition, past percentages refer to “most abnormal” abnormal ages 10–15 years and current percentages refer to individuals ≥5 years old

**p < 0.01, ***p < 0.001 (indicates statistically significant difference between groups in ADI-R past endorsed items); ^^*p* < 0.01, ^^^*p* < 0.001 (indicates statistically significant difference between groups in ADI-R current endorsed items)

#### Developing and maintaining relationships (symptom area A3; six ADI-R items)

No ADOS items were included in the symptom area of deficits in developing and maintaining relationships (A3). None of the ADI-R items met the study criteria as adequate discriminators between the groups, and results of *χ*^2^ tests of differences in proportion did not indicate any statistically significant differences between groups in the proportion of endorsed items (Table 4).

**Table 5:** Social communication items that met the criteria as adequate discriminators between ASD and ADHD

ADOS item	Mod. 3 item	Mod. 4 item	DSM-5 symptom domain
Quality of Social Overtures	B7	B9	Social-emotional reciprocity (a1)
Amount of Reciprocal Social Communication	B10	B12	Social-emotional reciprocity (a1)
Unusual Eye Contact	B1	B1	Non-verbal communication (a2)
Facial Expressions Directed to Examiner	B2	B2	Non-verbal communication (a2)

Items were defined as adequate discriminators if they were endorsed in >66 % of the ASD group *and* <33 % of the ADHD group

#### Stereotyped/repetitive speech, motor movements, or use of objects (symptom area B1; three ADOS items, six ADI-R items)

*χ*^2^ tests of differences in proportion indicated that two items on the ADOS, Stereotyped/Idiosyncratic Use of Words or Phrases and Hand and Finger and Other Complex Mannerisms, were endorsed significantly more frequently in the ASD group than the ADHD group (see Table 3). Although formal discrimination criteria were not applied to these items, Stereotyped/Idiosyncratic Use of Words or Phrases on the ADOS was endorsed in 71 % of the ASD group and 31 % of the ADHD group. On the ADI-R, one of the items, Stereotyped Utterances and Delayed Echolalia (item 33, ever), was endorsed significantly more frequently in the ASD group than the ADHD group (tests of differences in proportion; see Table 4).

#### Excessive adherence to routines, ritualized patterns, or resistance to change (symptom area B2; one ADOS item, four ADI-R items) and highly restricted, fixated interests (symptom area B3; one ADOS item, three ADI-R items)

For symptom area B2, none of the ADOS or the ADI-R items differed in the frequency of endorsement between the groups (*χ*^2^ tests of differences in proportion). For symptom area B3, Excessive Interest in or References to Unusual or Highly Specific Topics or Objects or Repetitive Behaviors on the ADOS was endorsed more frequently in the ASD group than the ADHD group, but there were no significant differences in the ADI-R items (tests of differences in proportion; see Tables 3 and 4).

#### Hyper-/hypo-reactivity to sensory input or unusual interest in sensory environment (symptom area B4; one ADOS item, three ADI-R items)

Unusual Sensory Interest in Play Material/Person on the ADOS was endorsed significantly more frequently in the ASD group than the ADHD group (tests of differences in proportion; see Table 3). There were no significant differences between the groups in the proportion of endorsed items on the ADI-R in this symptom area (tests of differences in proportion; see Table 4).

## Discussion

Results of this work highlight the diagnostic conundrum that clinicians often face when assessing verbally fluent, school-aged children for ASD [18, 36, 37, 56, 57]. Distinguishing a child with ASD from a child with ADHD (or other behavioral/psychiatric problems) in clinical settings can be challenging. Differential diagnosis in these cases is also very different from distinguishing a child with ASD from a typically developing child with no psychiatric history and/or no related parental concerns. In this study, we investigated parent-reported and clinician-observed ASD symptoms in a unique sample of children referred to ASD clinics who ultimately received diagnoses of ADHD without ASD and compared these children to a similarly referred group of children who ultimately received diagnoses of ASD. We explored these symptoms in a global (domain level) and detailed (item level) manner using scores from the ADOS and ADI-R, in order to provide both a dimensional and individual symptom profile. Overall, results suggest that many ASD symptoms were endorsed in some children with ADHD, which is consistent with previous studies [5, 15–17] and not surprising given that these children were referred due to ASD concerns. When looking at standard diagnostic cut-offs, combining the ADOS and ADI-R resulted in the highest specificity in the ADHD sample. However, when using the same combination of measures, sensitivity within the ASD sample decreased substantially. Using less stringent ADI-R ASD cut-offs (CPEA) [53], combined with standard ADOS ASD cut-offs resulted in the best combination of specificity within the ADHD sample and sensitivity within the ASD sample. However, it should be noted that CPEA criteria do not technically require the presence of RRBs, which would be required to make a DSM-5 clinical diagnosis.

On a dimensional level, children with ADHD scored significantly lower than children with ASD across all domains on the ADOS and in several domains on the ADI-R. However, on the ADI-R, parents reported that their children with ADHD displayed similar levels of current ASD symptoms, as well as an early age of onset. A relatively large proportion of children with ADHD also met cut-offs on several domains of the ADI-R algorithm. In contrast, domains of the ADOS were better at distinguishing between the diagnostic groups in this sample. On an individual item level, most ADOS items but only a few ADI-R items were endorsed more frequently in the ASD group. Moreover, four social communication items on the ADOS that fell within the DSM-5 domains of social-emotional reciprocity and non-verbal communicative behaviors were adequate at discriminating this unique group of children with ADHD from the children with ASD, but none of the social communication items on the ADI-R met study criteria for adequacy at discriminating between the ASD and ADHD groups. These results suggest that, when coded by a clinician during direct observation, there *are* differences in the quality and type of social impairment between children with ASD and children with ADHD that may be harder to differentiate based on parent report.

During direct observation (ADOS), symptoms in the RRB domain were seen more frequently in the ASD group than the ADHD group. Stereotyped/Idiosyncratic Use of Words or Phrases on the ADOS and Stereotyped Utterances and Delayed Echolalia on the ADI-R were the RRB symptoms most clearly associated with ASD in this study. On the other hand, a number of children with ADHD exhibited RRBs similar to children with ASD (at least based on parent report). This has implications for clinical practice as the specificity of these behaviors may be limited depending on the comparison group.

Although elevated scores on ASD measures in this sample of children with ADHD may suggest that actual ASD symptoms are common in ADHD [9, 10, 18], results of the clinician observation (ADOS) and the BEC non-ASD diagnoses suggest that there are important differences in the social communication behavior characteristic of each group that were perhaps not adequately captured by parent report. Elevated ASD symptom scores are more understandable in questionnaires, where parents interpret the meaning of certain questions, but elevations in an extensive, standardized parent interview are surprising. With the increasing amount of media attention on ASD, as well as the increasing number of services available to support individuals with ASD, parents may be more aware of and/or more likely to report ASD-like difficulties. At the same time, clinicians may weigh their impressions from the ADOS more heavily than the ADI-R in determinations of BEC, leading to non-ASD diagnoses when observations during the ADOS are not consistent with an ASD diagnosis, regardless of elevated ADI-R scores. Overall, it remains unclear whether elevations on the ADI-R are the result of parental over-reporting of ASD symptoms, misinterpreting behaviors as symptoms of ASD, or whether many children with ADHD truly exhibit social communication deficits similar to those seen in ASD. Nevertheless, these results underscore the need to recognize that social problems are *not* specific to ASD and that interventions to address social difficulties should not be dependent on an ASD diagnosis.

These results also suggest that care must be taken with regard to proposals of quick diagnoses based on abbreviated information [58], questionnaires [59], or diagnoses made through chart reviews [60]. Using solely chart reviews to determine a diagnosis may lead to misclassification of children with other psychiatric diagnoses, like ADHD, as our results highlight that ASD symptoms are often endorsed by parents [61]. Recently, Wall and colleagues [58] identified seven items from the ADI-R (past items) that distinguished ASD from typical development with >90 % accuracy: Comprehension of Simple Language, Reciprocal Conversation, Imaginative Play, Imaginative Play with Peers, Direct Gaze, Group Play with Peers, and Age when Abnormality First Evident [58]. Although only Reciprocal Conversation was mapped to DSM-5 diagnostic criteria for our sample [30], all of these items were endorsed in >60 % of our ADHD sample, with the exception of Comprehension of Simple Language (endorsed in 36 % of the ADHD sample) [58]. Thus, even if adequate differentiation between ASD and typically developing children is possible using abbreviated measures, the ability to distinguish between ASD and other childhood disorders may be minimal.

### Limitations

Our relatively small sample of children with ADHD represents a particular subset of children with ADHD: children whose parents had sufficient concern about ASD to bring them to an ASD-specialty clinic to determine whether a diagnosis of ASD was warranted. As such, these children are not representative of all children with ADHD. In addition, our group of children with ASD was deliberately selected from a much larger sample to be equivalent to the ADHD group in language level, age, and IQ and is therefore not representative of the whole ASD population. Future work should extend these results to samples that include individuals with lower language and intellectual abilities. In addition, this sample included relatively few females. Although this is consistent with the gender discrepancy seen in ASD samples, results of this study may not be characteristic of females with ASD and/or ADHD. Future studies should explore the unique symptom presentations in females with ASD and/or ADHD. Last, we could not rule out ADHD in our sample of children with ASD, although children with formal comorbid diagnoses of ADHD were excluded.

## Conclusions

There are many challenges in assessing verbally fluent, school-age children referred for possible ASD. Although some social communication symptoms measured by standard observations distinguished children with ASD from children with ADHD, domain cut-off scores on a detailed parent interview were less useful in diagnostic discrimination. Results of this study call for additional research exploring the prevalence, quality, intensity, and trajectories of social communication impairments in children with ADHD compared to children with ASD, in order to provide insights into the boundaries and overlaps between these disorders across development. Furthermore, detailed phenotyping of ASD-like traits in these children could provide insight into studies exploring the neurobiological [33, 34] and genetic [32] underpinnings of these disorders, both when they occur alone and together. In addition, recognition of the nature of social deficits in children with ADHD, regardless of whether they also meet criteria for ASD, is essential to tailor effective treatments [62–64]. Future research in children with ADHD not referred for possible ASD is also clearly warranted in order to understand ASD symptoms in a more representative sample of children with ADHD.

## Appendix

**Table 6:** ANOVA results comparing groups on ADOS and ADI-R

	ADHD	ASD			
	(*n* = 48)	(*n* = 164)			
	Mean (SD)	Mean (SD)	*F*	*df*	*p* value
ADOS domains^a^					
CSS SA	3.3 (2.0)	6.4 (2.4)	66.80	1, 210	<0.001
CSS RRB	3.3 (2.5)	7.0 (2.5)	80.60	1, 210	<0.001
CSS overall	2.7 (1.6)	6.5 (2.5)	99.51	1, 210	<0.001
ADOS factors^b^					
Basic soc	1.2 (1.2)	3.2 (2.1)	41.16	1, 201	<0.001
Interaction quality	1.8 (1.3)	3.6 (1.9)	38.19	1, 210	<0.001
RRBs	0.6 (0.8)	2.7 (1.9)	53.26	1, 210	<0.001
ADI-R algorithm domains					
Social total	12.1 (7.1)	16.1 (7.0)	11.43	1, 200	<0.01
Communication total	10.4 (6.3)	13.7 (5.3)	12.24	1, 200	<0.01
RRB-ADI total	4.6 (3.2)	6.0 (3.0)	8.21	1, 200	<0.01
ADI-R current domains					
SC total	13.0 (7.5)	13.7 (7.1)	0.33	1, 184	n.s.
RSM total	2.9 (2.5)	4.1 (2.7)	6.66	1, 193	n.s.
IS total	5.6 (3.4)	5.2 (3.0)	0.34	1, 127	n.s.

*ADHD* attention deficit/hyperactivity disorder, *ASD* autism spectrum disorder, *CSS* calibrated severity score, *df* degrees of freedom, *IS* insistence on sameness, *n.s.* not significant (*p* value >0.01), *RRBs* restricted repetitive behaviors on ADOS, *RRB-ADI* restricted repetitive behaviors on ADI-R, *RSM* repetitive sensory motor, *SA* social affect, *SC* social communication, *SD* standard deviation

^a^Includes both modules 3 and 4

^b^See text for details based on Bishop, Havdahl, Huerta, and Lord (in prep); see text for ANCOVA results

## Abbreviations

ADHD: Attention Deficit/Hyperactivity Disorder
ADI-R: Autism Diagnostic Interview-Revised
ADOS: Autism Diagnostic Observation Schedule
ANCOVA: analysis of covariance
ANOVA: analysis of variance
APA: American Psychological Association
ASD: Autism Spectrum Disorder
Basic SOC BEC: Basic Social Communication factor (ADOS) best estimate clinical diagnosis
CADB: Center for Autism and the Developing Brain
CELF: Clinical Evaluation of Language Fundamentals
CPEA: Collaborative Program of Excellence in Autism
CSS: Calibrated Severity Scores
DAS: Differential Ability Scales
DSM-5: Diagnostic and Statistical Manual of Mental Disorders, Fifth Edition
DSM-IV-TR: Diagnostic and Statistical Manual of Mental Disorders, Fourth Edition, Text Revision
IRB: Institutional Review Board
IS: Insistence on Sameness
NVIQ: Non-Verbal IQ
PPVT: Peabody Picture Vocabulary Test
RDoC: Research Domain Criteria
RRB: Restricted and Repetitive Behavior
RSM: Repetitive Sensory Motor
SA: Social Affect
SC: Social Communication domain (ADI-R)
UCDDC: University of Chicago Developmental Disorders Clinic
UMACC: University of Michigan Autism and Communication Disorders Center
VABS: Vineland Adaptive Behavior Scales
VIQ: Verbal IQ
WAIS: Wechsler Adult Intelligence Scale
WASI: Wechsler Abbreviated Scale of Intelligence
WISC: Wechsler Intelligence Scale for Children
WPPSI: Wechsler Preschool and Primary Scale of Intelligence
